# New Preservative for Animal Substances

**Published:** 1843-11

**Authors:** 


*New Preservative for Animal Substances.*—A French physician has addressed a paper to the Academy of Sciences on the power of a *syrup of iron* to preserve animal substances unchanged. This syrup is a combination of sugar and iron which does not decompose, crystallise, or ferment, at any temperature. Meats kept in this syrup diminish very little in weight, resist the most active putrefactive agency, and on being washed in cold water resume their original volume and appearance as from animals newly killed.—*Ibid.*

